# Testicular Torsion Appearance and Diagnosis on Computed Tomography of the Abdomen and Pelvis: Case Report

**DOI:** 10.5811/cpcem.2022.2.55315

**Published:** 2022-04-21

**Authors:** Graham S. Stephenson, Mark I. Langdorf

**Affiliations:** University of California, Irvine, Department of Emergency Medicine, Orange, California

**Keywords:** testicular torsion, whirl sign, abdominal pain

## Abstract

**Introduction:**

Testicular torsion, or the twisting of the spermatic cord compromising blood flow to the testis, is a urologic emergency with the potential to cause infertility in male patients. The diagnosis may be clinical or confirmed using imaging, with ultrasound being the modality of choice.

**Case Report:**

We present a case of right lower quadrant pain with radiation to the groin and right scrotum in a young male. A computed tomography of the abdomen and pelvis was ordered to assess for appendicitis, which showed a “whirl” sign on the inferior periphery of the images near the scrotum. The finding was not appreciated during the emergency department visit and the patient was discharged home. He returned 48 hours later due to continued pain and was ultimately diagnosed with testicular torsion via ultrasound and surgical pathology.

**Conclusion:**

This is the first reported case to our knowledge identifying “whirl” sign for the diagnosis of testicular torsion. This finding was not appreciated by multiple clinicians during the initial patient presentation, highlighting the uncommon nature of the finding.

## INTRODUCTION

The internal and external spermatic arteries travel through the spermatic cord to supply blood to the testicles. Testicular torsion, or the twisting of the testes on the spermatic cord, impedes this supply and is a urologic emergency. Failure to promptly reduce torsion may result in infertility from ischemic loss of germ cells or the generation of anti-sperm antibodies.^1^ Although most common in neonates and pre-pubertal males, torsion may occur at any age with nearly 40% outside typical demographics.^2^ In the United States, testicular torsion occurs 5.9 times per 100,000 males ages 1–17 years, and 1.3 per 100,000 over the age of 18.^3^

A concerning physical exam is sufficient to make the diagnosis of testicular torsion. Presenting symptoms include a firm and tender testicle, abnormal testicle lie (horizontal or high riding), loss of the cremasteric reflex, nausea and vomiting, and lower abdominal pain.^4^ Symptoms often present after physical activity or minor trauma, although younger patients may be startled from sleep due to nocturnal cremasteric contraction. Testicular torsion during adolescence will frequently occur within the tunica vaginalis, causing a “bell clapper” deformity where the affected testicle has an abnormal transverse lie in the standing patient.^5^

When clinical exam is equivocal, imaging is highly sensitive and can confirm the diagnosis. Ultrasound with color Doppler is the test of choice with sensitivity and specificity of 82% and 100%, respectively.^6^,^7^,^8^ Ultrasound findings concerning for torsion include direct visualization of twisted cord with a “whirl” sign, diminished blood flow on Doppler, abnormal echo texture of the affected side, reactive hydrocele, or scrotal wall thickening and hyperemia. Objectively, computed tomography (CT) is an inferior imaging modality for evaluation of suspected testicular torsion with data for efficacy limited to small-scale experimental perfusion studies.^9^,^10^

We present a case of right-sided testicular torsion where a CT of the abdomen and pelvis was obtained for suspicion of acute appendicitis. The visible “whirl” sign at the periphery of the abdominopelvic CT, representing the twisted spermatic cord, was not appreciated by the radiologist or the emergency physician (EP). Testicular torsion was later confirmed by ultrasound 48 hours after the initial presentation. To our knowledge, this is the first reported case of “whirl” sign representing testicular torsion seen on CT, and it highlights the importance of critical evaluation of imaging in concert with presenting symptoms.

## CASE REPORT

A 21-year-old man with no previous medical history presented to the emergency department (ED) with sudden right lower quadrant (RLQ) pain for 3.5 hours. The pain was constant and radiated to his scrotum, with reported pain when the scrotum was touched. He had the urge to defecate at onset, with increased flatulence, and multiple episodes of diarrhea. He denied frequency, urgency, penile discharge, and dysuria. He was not sexually active.

The presenting vital signs were blood pressure of 128/71 millimeters of mercury, heart rate of 92 beats per minute, respiratory rate of 16 breaths per minute, temperature of 36.6° Celsius, and oxygen saturation of 100% on room air. His documented exam included mild RLQ tenderness to palpation without rebound or guarding. His physical exam was otherwise marked as normal, with no genitourinary exam documented.

The physician’s assistant ordered tests from triage, including a urinalysis, complete blood count with differential, comprehensive metabolic panel, intravenous (IV) fluids, pain control, and a CT of the abdomen and pelvis with IV contrast. The patient was given 15 milligrams (mg) of ketorolac, 4 mg of morphine sulphate, and 1 liter (L) of normal saline. Laboratory tests showed white blood cell (WBC) count of 11.6 x 10^9^ cells/L (reference range [RR] 3.8–11 x 10^9^ cells/L), normal serum electrolytes, urinalysis with 3 WBCs per high powered field (hpf) (0–5/ hpf), negative leukocyte esterase and nitrite (RR negative), and no bacteria (RR none/hpf). Urine polymerase chain reaction tests for gonorrhea and chlamydia resulted negative the following day.

**Image 1:**
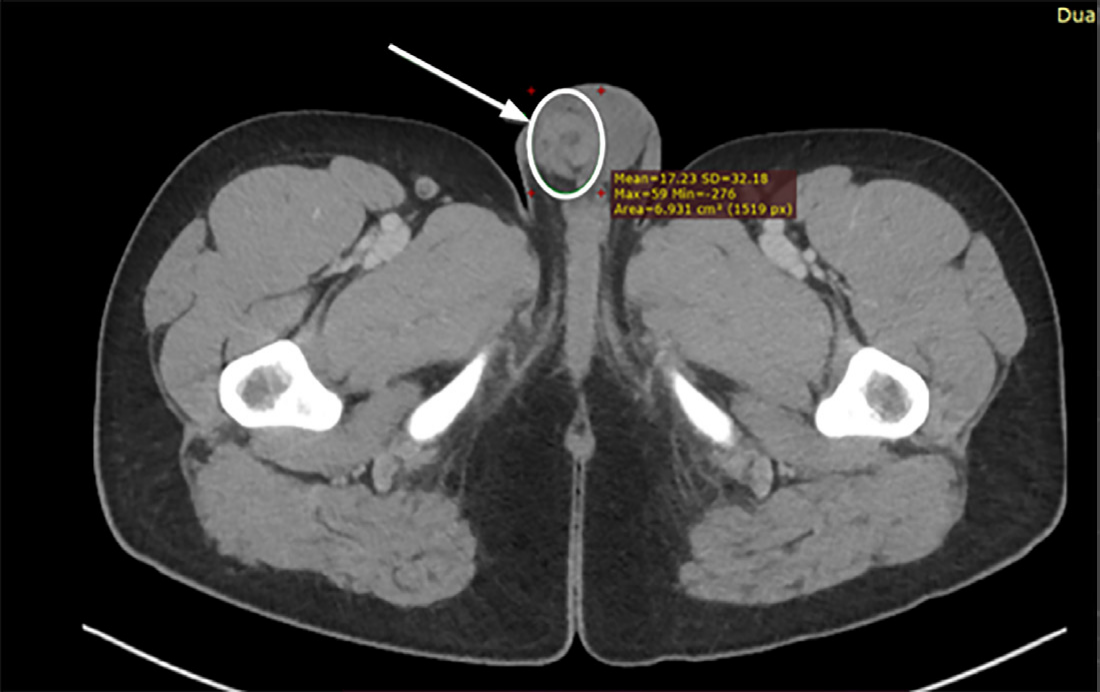
Axial computed tomography of the abdomen and pelvis showing “whirl” sign in the right scrotum at the inferior periphery of the image suggestive of testicular torsion. The white arrow and circle highlight this finding.

**Image 2:**
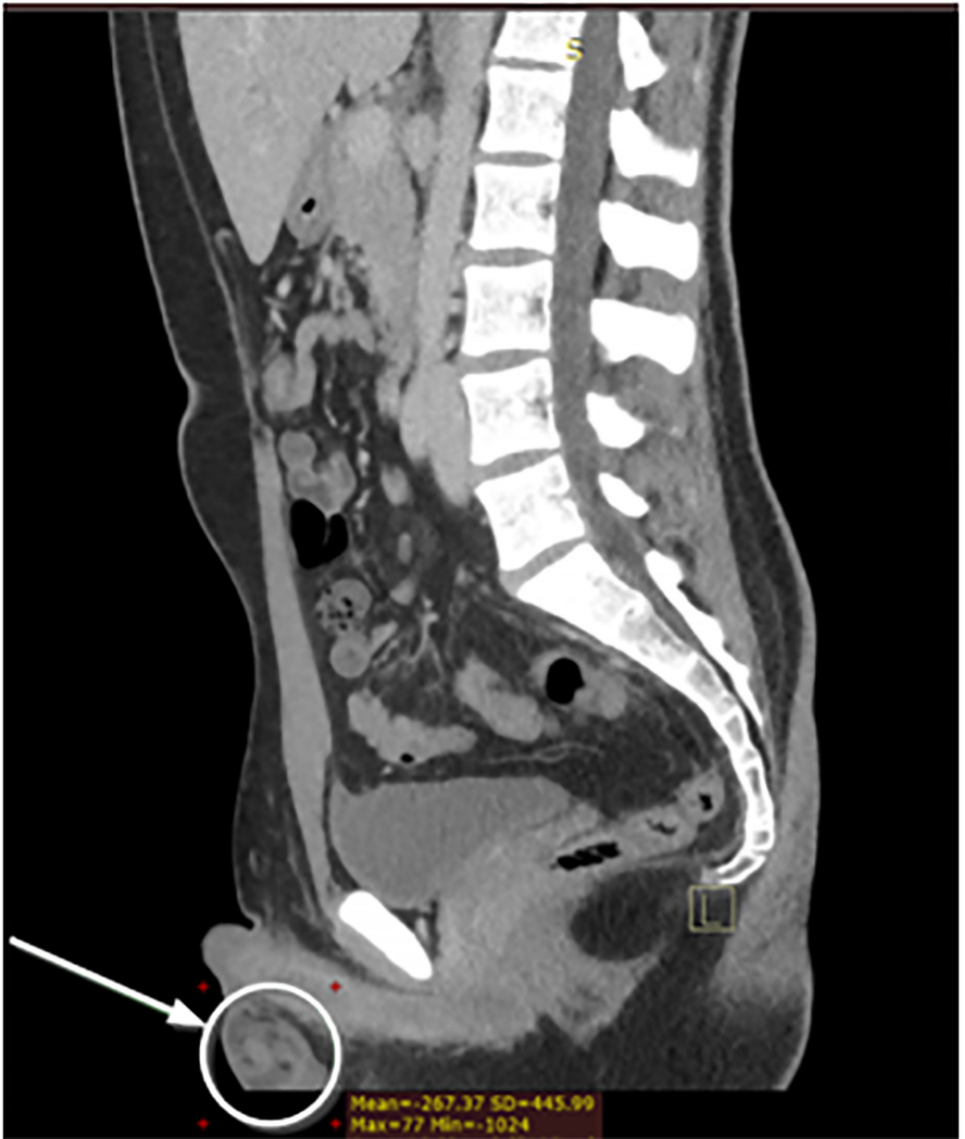
Right parasagittal computed tomography of the abdomen and pelvis showing “whirl” sign consistent with testicular torsion in the right scrotum at the inferior periphery of the image. The white arrow and circle highlight this finding.

A CT of the abdomen and pelvis with IV contrast was ordered to evaluate for appendicitis. The radiology report of the abdomen and pelvis (Image 1, Image 2) was as follows: “unremarkable CT of the abdomen and pelvis. The appendix is not discretely identified; however, no inflammatory changes are seen in the right lower quadrant to suggest acute appendicitis.” There was no comment on the scrotum in the report. The patient’s symptoms were documented as improved upon re-evaluation and he was discharged home.

**CPC-EM Capsule**

What do we already know about this clinical entity?

*Testicular torsion is a urologic emergency. Although primarily a clinical diagnosis, ultrasound is often used to confirm clinical suspicion*.

What makes this presentation of disease reportable?

*We present a case of abdominal pain in a young male. Computed tomography (CT) demonstrated a “whirl” sign of the spermatic cord later confirmed to be testicular torsion*.

What is the major learning point?

*This is the first report detailing abnormal image findings on CT that support the diagnosis of testicular torsion*.

How might this improve emergency medicine practice?

*“Whirl” sign on CT should greatly raise the clinician’s concern for underlying ischemia, including during evaluation of testicular pain*.

The patient’s symptoms continued, and roughly 48 hours later he was seen in urgent care. There, he had a testicular ultrasound showing no arterial or venous flow in the right testicle. He was sent to the same ED, and re-evaluation was documented as “phallus circumcised without lesions. Right testis is high riding, exquisitely tender and mildly edematous. Left testis is normal and non-tender.” A urologist was consulted for emergent right orchiectomy and left orchiopexy. The operative report documented a necrotic right testis, and the surgeon noted 540 degrees (1.5 rotations) of spermatic cord torsion. Pathology report confirmed, “hemorrhagic infarct of the testicular and epididymal parenchyma consistent with torsion. Spermatic cord with vascular congestion. No evidence of malignancy.”

## DISCUSSION

Testicular torsion stems from the twisting of the testes on the spermatic cord when there is inadequate fixation of the testes on the tunica vaginalis. Although gross testicle and sperm viability begins to decline at eight hours after torsion, this progresses over the next 18–24 hours, and some salvage may occur during or after this period.^11^ Therefore, prompt diagnosis and treatment are critical to preserve fertility. When exam alone is not diagnostic, imaging should be done. In this case, the “whirl” sign was visible at the periphery of the CT.

The “whirl sign” is evident on CT in other body organs that twist, including the fallopian tube, ovary, and mesentery (cecal or sigmoid volvulus).^12^ It is diagnostic on high-resolution ultrasound of the spermatic cord and fallopian tube.^13^,^14^ High-resolution ultrasound may detect torsion with up to 96% sensitivity and 99% specificity. The CT appearance here mimics these known examples of twisting organs. We report this case to encourage EPs and radiologists to scrutinize the periphery of the CT to potentially improve the diagnosis of testicular torsion.

## CONCLUSION

As with other bodily organs that can twist, the “whirl” sign may be seen on CT in cases of testicular torsion as demonstrated in this case. Although ultrasound is the diagnostic study of choice, CT can provide evidence supporting the diagnosis of testicular torsion and should not be ignored during the evaluation of patients with genitourinary pain.
